# The Vital Role of Family Physicians in the Screening and Early Detection of Lynch Syndrome: A Case Report

**DOI:** 10.7759/cureus.102547

**Published:** 2026-01-29

**Authors:** José Campelos, Maria Gomes, Alexandra Campos, Maria Cardoso, Mariana Lopes

**Affiliations:** 1 Family Medicine, Valflores Family Health Center, São José Local Care Unit, Lisbon, PRT

**Keywords:** family medicine, genetic testing, genogram, hereditary cancer, lynch syndrome, mlh1

## Abstract

Lynch syndrome (LS) is an autosomal dominant disorder associated with an increased risk of colorectal, endometrial, and other malignancies. This report describes the case of a 53-year-old female patient with a significant family history of colorectal cancer. She presented with metrorrhagia and iron deficiency anemia. Given her strong familial cancer history, besides the transvaginal ultrasound, a colonoscopy was performed despite a recent normal result. The new colonoscopy revealed a vegetative and ulcerated neoplasm at the rectosigmoid junction, confirmed as adenocarcinoma by biopsy. Genetic testing identified a pathogenic *MLH1* gene variant (c.2041G>A p.Ala681Thr), confirming LS. The patient was referred for Oncology, Gastroenterology, Surgery, and Genetics consultations. A segmental colectomy was performed along with prophylactic surgery, including hysterectomy and salpingo-oophorectomy. This case highlights the vital role of family physicians in recognizing hereditary cancer syndromes, utilizing genograms to expedite diagnosis, and ensuring proper screening for associated extracolonic malignancies in affected families.

## Introduction

Lynch syndrome (LS), also known as hereditary non-polyposis colorectal cancer (HNPCC), is the most common hereditary colorectal cancer syndrome, accounting for 3-5% of colorectal carcinomas [1-3]. LS is caused by germline mutations in DNA mismatch repair (MMR) genes, most frequently *MLH1*, *MSH2*, *MSH6*, and *PMS2*, which lead to microsatellite instability and a markedly increased risk of developing various malignancies [1,4-8]. In addition to colorectal cancer, LS significantly increases the lifetime risk of endometrial, ovarian, gastric, small intestine, hepatobiliary tract, urinary tract, and brain cancers [2-4].

Timely recognition of LS is essential, as early diagnosis allows for life-saving surveillance, risk-reducing surgeries, and tailored management strategies that can significantly lower cancer-related morbidity and mortality [4,5,7]. Recent research has emphasized the importance of personalized risk stratification based on the specific MMR gene affected, as well as the potential role of immunotherapy and vaccines in the prevention and treatment of LS-associated malignancies [1,9].

Lifestyle factors have been identified as modifiable risk contributors that may influence cancer development in LS carriers, underscoring the importance of holistic patient management [10]. Despite advances in genetic testing and clinical guidelines, LS remains underdiagnosed, often due to gaps in family history assessment and delayed recognition of hereditary patterns [1,4]. 

Family physicians play a pivotal role in identifying patients at risk for LS, particularly by constructing detailed family histories and genograms that reveal patterns of early-onset or multiple cancers within families. This case report illustrates the critical importance of primary care in the early detection and multidisciplinary management of LS.

## Case presentation

A 53-year-old woman presented to her family physician with complaints of heavy and irregular metrorrhagia over the past several months, accompanied by fatigue and lightheadedness. She had a medical history of hypertension, dyslipidemia, and obesity, for which she was taking a fixed-dose combination of perindopril and amlodipine (5 mg + 5 mg daily). She denied smoking, alcohol consumption, or illicit drug use.

**Figure 1:**
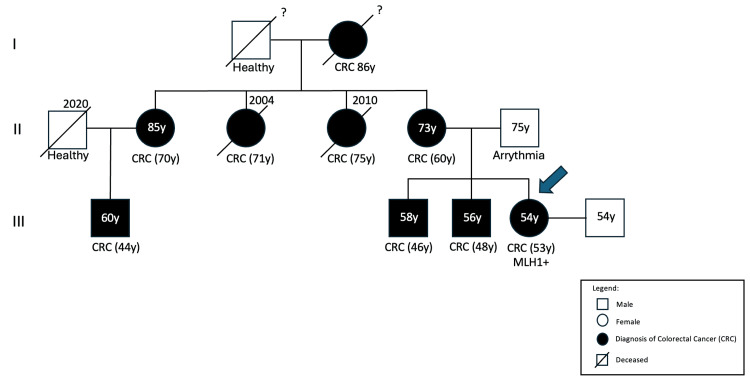
Genogram CRC: colorectal cancer; y: years

Her family history was remarkable for multiple cases of early-onset colorectal cancer. Her mother, two brothers, three maternal aunts, and a cousin had all been diagnosed with colorectal cancer, some under the age of 50. A detailed genogram was constructed, revealing a clear autosomal dominant inheritance pattern suggestive of a hereditary cancer syndrome (Figure 1).

**Figure 2:**
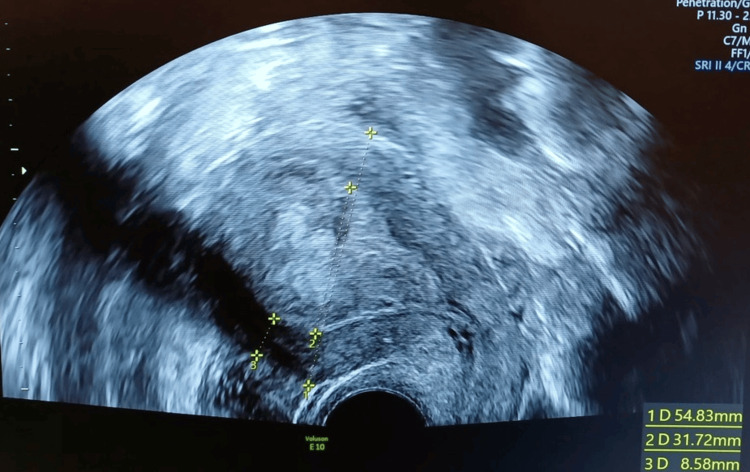
Transvaginal ultrasound Line 2: endometrial thickening

Initial laboratory tests showed iron-deficiency anemia with a hemoglobin level of 8.3 g/dL (reference interval: 12-16 g/dL). A transvaginal ultrasound was performed to investigate the cause of metrorrhagia, which revealed an endometrial thickening (Figure 2).

**Figure 3:**
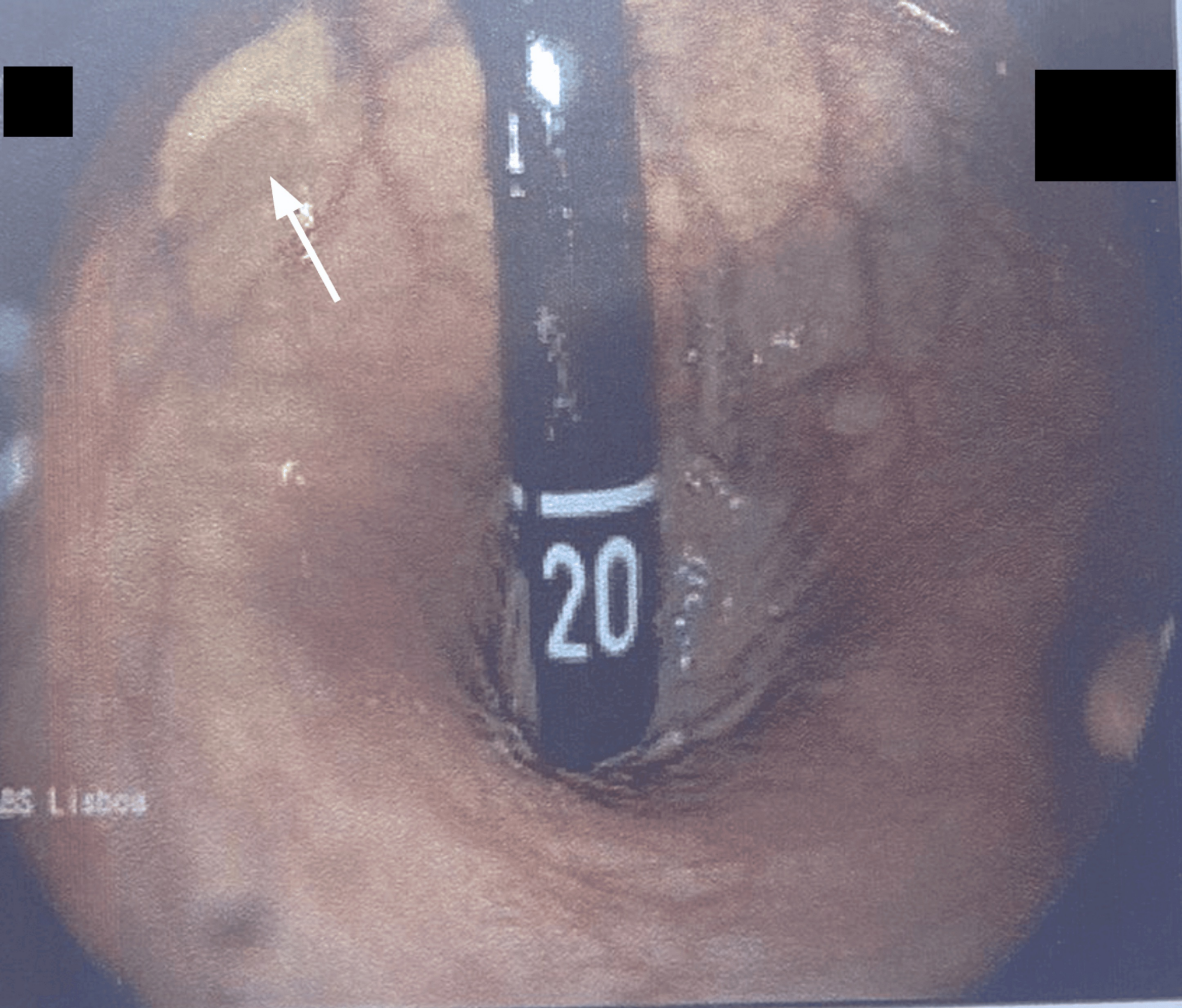
Colonoscopy (rectal retroflexion) showing an ulcerated mass (arrow) at the rectosigmoid junction

**Figure 4:**
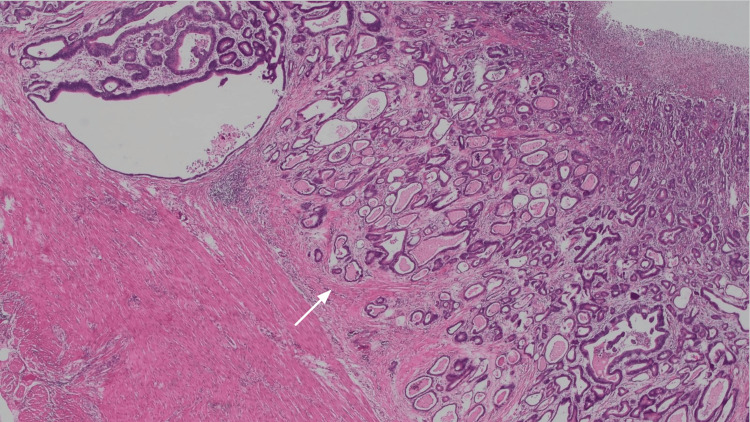
Histopathological analysis of the resection specimen Arrow: Adenocarcinoma

Despite having undergone a normal colonoscopy two years prior, her strong family history prompted the family physician to request an early repeat colonoscopy. The new colonoscopy identified a vegetative, ulcerated mass at the rectosigmoid junction (Figure 3). Histopathological analysis of the biopsy confirmed a diagnosis of moderately differentiated adenocarcinoma (Figure 4). The patient was promptly referred to Oncology, Gastroenterology, Surgery, and Genetics for comprehensive evaluation.

Genetic testing revealed a heterozygous pathogenic variant in the *MLH1* gene (c.2041G>A p.Ala681Thr), confirming the diagnosis of LS.

While guidelines generally state that young patients with *MLH1* mutations should undergo extensive surgical resection due to the high risk of metachronous tumors, the decision should be individualized [4]. At 53 years of age, this patient sits in a clinical transition zone, where the functional morbidity and decreased quality of life associated with an extensive approach often outweigh the long-term benefits. Because the cumulative risk of a metachronous lesion is lower at this age compared to younger patients, the surgical team performed a segmental colectomy. This was combined with a prophylactic total hysterectomy and bilateral salpingo-oophorectomy to address the elevated risk of endometrial and ovarian cancers. This integrated approach was deemed appropriate given that the patient is beyond childbearing age.

The patient recovered well postoperatively and was enrolled in a tailored surveillance program. Cascade genetic testing was offered to her first-degree relatives, and those who tested positive were integrated into appropriate cancer prevention and screening pathways.

## Discussion

LS is the most common hereditary colorectal cancer syndrome, characterized by germline mutations in DNA MMR genes such as *MLH1, MSH2, MSH6*, and *PMS2* [1]. This genetic defect leads to microsatellite instability and significantly increases the risk of colorectal, endometrial, and other extracolonic malignancies [1,2]. The lifetime risk of colorectal cancer in LS patients can approach 70%, while the risk of endometrial cancer can reach up to 60%, particularly in women with *MLH1* or *MSH2* mutations [2,9].

In this case, the patient’s early identification of a hereditary cancer pattern through a detailed family history and genogram was crucial for expedited diagnosis. Family physicians are often the first to detect these hereditary patterns, playing a central role in initiating appropriate genetic evaluations and referrals [4]. Despite a normal colonoscopy just two years prior, the strong family history correctly raised suspicion, prompting an early repeat colonoscopy that revealed the colorectal tumor. This emphasizes the importance of personalized screening intervals based on genetic risk rather than routine recommendations [4,10]. 

The patient’s management was consistent with current best practices, which recommend that women with LS undergo consideration of prophylactic hysterectomy and bilateral salpingo-oophorectomy due to the high risk of gynecologic cancers [9]. This combined surgical approach addresses both existing malignancy and future cancer risk, offering significant survival benefit.

Cascade genetic testing is essential in LS to identify at-risk relatives who may benefit from intensified surveillance and preventive strategies [1,10]. In this case, family counseling and genetic testing were offered to first-degree relatives, enabling early intervention and personalized care for those who tested positive. 

This case highlights the importance of the family physician's vigilance in recognizing potential hereditary cancer syndromes, the critical value of genograms in primary care, and the need for dynamic cancer screening tailored to genetic risk profiles.

## Conclusions

LS is a prevalent hereditary cancer syndrome that significantly increases the risk of colorectal and extracolonic cancers. Early recognition through detailed family history assessment and genogram construction in primary care is vital for timely diagnosis and intervention. This case demonstrates the crucial role of family physicians in identifying hereditary cancer patterns, facilitating appropriate genetic testing, and coordinating multidisciplinary care. Personalized surveillance and risk-reducing strategies, including prophylactic surgery, improve patient outcomes and reduce cancer-related morbidity. Cascade testing and counseling of relatives are essential components of comprehensive LS management, enabling prevention and early detection in at-risk family members.
